# Convergency and Stability Responses of Bacterial Communities to Salinization in Arid and Semiarid Areas: Implications for Global Climate Change in Lake Ecosystems

**DOI:** 10.3389/fmicb.2021.741645

**Published:** 2022-01-04

**Authors:** Yang Hu, Xingyu Jiang, Keqiang Shao, Xiangming Tang, Boqiang Qin, Guang Gao

**Affiliations:** Taihu Laboratory for Lake Ecosystem Research, State Key Laboratory of Lake Science and Environment, Nanjing Institute of Geography and Limnology, Chinese Academy of Sciences, Nanjing, China

**Keywords:** climate change, convergency, nutrient enrichment, salinization, stability

## Abstract

Climate change has given rise to salinization and nutrient enrichment in lake ecosystems of arid and semiarid areas, which have posed the bacterial communities not only into an ecotone in lake ecosystems but also into an assemblage of its own unique biomes. However, responses of bacterial communities to climate-related salinization and nutrient enrichment remain unclear. In September 2019, this study scrutinized the turnover of bacterial communities along gradients of increasing salinity and nutrient by a space-for-time substitution in Xinjiang Uyghur Autonomous Region, China. We find that salinization rather than nutrient enrichment primarily alters bacterial communities. The homogenous selection of salinization leads to convergent response of bacterial communities, which is revealed by the combination of a decreasing β-nearest taxon index (βNTI) and a pronounced negative correlation between niche breadth and salinity. Furthermore, interspecific interactions within bacterial communities significantly differed among distinct salinity levels. Specifically, mutualistic interactions showed an increase along the salinization. In contrast, topological parameters show hump-shaped curves (average degree and density) and sunken curves (modularity, density, and average path distance), the extremums of which all appear in the high-brackish environment, hinting that bacterial communities are comparatively stable at freshwater and brine environments but are unstable in moderately high-brackish lake.

## Introduction

In arid and semiarid areas, lake ecosystems are typically unproductive, and external disturbances dominate in-lake processes, making these systems ideal sentinels of environmental disturbance. As global climate change has intensified over the past several decades, arid and semiarid areas have been characterized by a negative moisture balance with evaporation exceeding precipitation (Sered et al., 2011). Consequently, the decline in water volume has caused salinization to be the most concerning effect on lake ecosystems (Slama et al., 2020; Ondrasek and Rengel, 2021). Concurrently, nutrient enrichment is also regarded as another most urgent environmental problem in arid and semiarid areas. Since implementation of the Great Western Development Strategy, a burst of anthropogenic activities has severely intensified in arid and semiarid areas in China (Liu et al., 2016, 2019). Increasing river flow driven by climate change potentially transports nutrients from the agricultural field and grassland on the catchment scale into lake ecosystems, which promotes nutrient loading (Charlton et al., 2018). In the context of climate change, the combination of salinization and nutrient enrichment renders the biotic communities of lake ecosystems in arid and semiarid areas not just as an ecotone but rather an assemblage with unique biomes and biotic processes (Lozupone and Knight, 2007; Tang et al., 2021). Thus, a flurry of research about the responses of lake ecosystems to climate change has led to an increasing interest in how communities respond to salinization and nutrient enrichment in arid and semiarid areas.

Relative to the macroorganisms, bacterial communities are extremely sensitive to environmental disturbances, whose change often integrates the biogeochemical processes of lake ecosystems (Rofner et al., 2017). Therefore, the dynamics of bacterial communities is critical to understanding the responses of lake ecosystems to climate-related disturbances. A global survey of natural environments has revealed the roles of salinization in determining the biogeographical patterns of bacterial communities (Lozupone and Knight, 2007). It is in arid and semiarid areas that the filtering effects of salinization are especially pronounced (Tang et al., 2012; Bencherif et al., 2015; Zhang et al., 2019; Kivistik et al., 2020). By inducing high osmotic pressure and intracellular ion concentrations, salinity is toxic to the growth of most freshwater bacterial individuals (Rath et al., 2019a,b) and their degradation ability (Chen et al., 2020; Zhang et al., 2020). Therefore, salinization threatens bacterial abundance, diversity, and functional metabolism (Yang et al., 2016; Lindsay et al., 2019). Furthermore, by enhancing the deterministic processes of environmental filtering, salinization induces a turnover of bacterial communities dominated by from halophobic to halophilic bacteria (Tang et al., 2021). When nutrient enrichment is taken into account, however, the stress of salinization on bacterial communities is alleviated. For instance, high nutrients can mitigate the constraining effects of salinity on bacterial energy-generating pathways (Yue et al., 2019). Consequently, nutrient enrichment decreases the deterministic processes and increases stochastic processes in bacterial communities (Tang et al., 2021). In this case, the uncertainty of bacterial community dynamics is promoted under simultaneous salinization and nutrient enrichment.

The stability is critical to predict the bacterial community dynamics (Pettersson et al., 2019). For decades, the community stability is considered to be primarily controlled by biodiversity and complexity. However, recent perspectives argued that the topology of interspecific interactions regulates community stability (Coyte et al., 2015; Qian and Akcay, 2019). In nature, bacteria tend to live together to form complex networks, which are mainly connected by antagonistic and mutualistic interactions. The antagonistic interactions exclude species from the community and result in a loss of biodiversity (Becker et al., 2012; Coyte et al., 2015; Ratzke et al., 2020), such that antagonism is expected to destabilize the bacterial community based on the complex-stability debate. However, this expectation has been overthrown by the theoretical work, which highlights that competition could benefit community stability (Gonze et al., 2017; Tu et al., 2019; Stone, 2020). To reconcile these opposite perspectives, several underlying mechanisms have been proposed, such as non-linear responses and saturating effects (Butler and O’Dwyer, 2018). Furthermore, how strongly and tightly individual bacteria link together also reflects community stability (Chen and Wen, 2021; Price et al., 2021). However, the topological properties of interspecific interactions of bacterial communities under climate change remains unknown. Therefore, there is a pressing need to identify the overarching patterns of biotic interactions in bacterial communities. Specifically, upon which members do each species rely? With which members do they compete, and how do these interactions change with climate change-driven disturbance?

Ultimately, we hypothesized that climate-related disturbances induced profound effects on the bacterial communities with imbalanced influences of salinization and nutrient enrichment. To verify this, we applied a space-for-time substitution (SFT) approach, which is one of the most commonly encountered techniques in ecology to extrapolate a temporal trend. It is noteworthy that there is a premise to use SFT: biotic communities should be extensive homologous in their past history which means the environmental disturbances mainly regulate the community assembly, but not initial conditions and priority effects (Wogan and Wang, 2018; Damgaard, 2019). Thus, we chose four adjacent lakes (approximately 30 km apart) which are similar in life history, and differ only in saline and nutrient environments. Based on the above hypothesis, we addressed the following questions: (1) Which climate-related disturbance primarily regulates the bacterial community dynamics? (2) What are the adaptive strategies of bacterial communities? (3) How does bacterial community stability respond to environmental disturbances? By addressing these issues, we attempt to provide new insights into the future responses of bacterial communities in relation to climate change.

## Materials and Methods

### Overview of the Study Area

**FIGURE 1:**
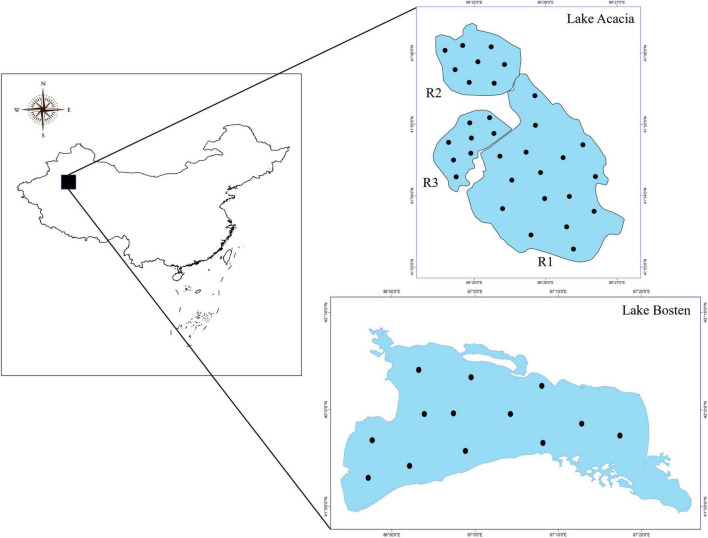
Location of the sampling sites.

Four adjacent lakes were chosen in the Xinjiang Uyghur Autonomous Region, China (Figure 1). Lake Bosten (86°40′–87°26′ E and 41°56′–42°14′ N) is the largest inland freshwater lake in China, and is located in the Yanqi Basin in the southern piedmont of the Tianshan Mountains. Lake Acacia (86°24′–86.27′ E and 41°53′–41°56 N) is located to the western side of Lake Bosten (approximately 30 km away), and consists of three isolated son lakes (R1, R2, and R3). For the last 10 years, local meteorological data showed the mean annual temperature was 8.6°C, the mean annual precipitation was 50–80 mm, and the annual mean evaporation was higher than 2,000 mm. Recent evidence has demonstrated that the mean annual temperature has risen by approximately 1.4°C over the past 50 years (Li et al., 2021).

### Field Sampling and Physico-Chemical Parameters Measurement

A total of 45 surface water samples (13, 16, 8, and 8 for Lake Bosten, R1, R2, and R3, respectively) were collected with a 5 L Schindler sampler in September 2019. A 500 mL subsample for 16 S rRNA gene analysis was filtered onto 0.22 μm polycarbonate membranes (47 mm diameter, Millipore) under a vacuum pressure of < 15 mm Hg. During transport back to the laboratory, the filters were stored at −20°C in a vehicle-mounted freezer; subsequently they were stored at −80°C in the laboratory for DNA extraction. The remaining water samples were transported to the laboratory for immediate further analysis. *In situ* measurements of water depth (WD), temperature (Temp), salinity, and dissolved oxygen (DO) were measured by a multiparameter water quality sonde (YSI 6600v2; United States). Five chemical parameters were measured upon return to the laboratory according to the standard methods described previously (Jin and Tu, 1990): total nitrogen (TN), total phosphorus (TP), ammonium (NH_4_^+^–N), dissolved organic carbon (DOC), and chemical oxygen demand (COD).

### DNA Extraction, Sequencing, and Data Processing

Crude DNA extracts were extracted by the E.Z.N.A^®^ Cycle-Pure kit (Omega Bio-Tek, Inc., Norcross, GA, United States). The V4 region of the 16 S rRNA gene was amplified using the primers 515F (5′-GTGYCAGCMGCCGCGGTAA-3′) and 806R (3′-GGACTACNVGGGTWTCTAAT-5′) (Sáenz et al., 2019). Unique barcodes were inserted into the primers to distinguish each sample. Polymerase chain reaction (PCR) amplification was performed in a 50-μL reaction mixture, containing 5 μL of 10 × PCR buffer, 4 μL of MgCl_2_ (25 mmol L^–1^), 0.5 μL of each primer (10 μmol L^–1^ each), 30 ng of quantified template DNA measured by PicoGreen dsDNA Assay Kit (Invitrogen, Carlsbad, CA), and 0.4 μL of *Taq* polymerase (5 U μL^–1^; Fermentas, United States) (Ahn et al., 1996). PCR cycling was conducted in a thermocycler (Applied Biosystems Veriti Thermal Cycler) by a touchdown program: denaturation at 94°C for 5 min, 11 cycles of denaturation at 94°C for 1 min, annealing at 65°C for 1 min (temperature was decreased by 1°C every cycle until 55°C was reached), and extension at 72°C for 1 min. Nineteen additional cycles were performed at an annealing temperature of 55°C, followed by a final extension at 72°C for 10 min. To reduce PCR-induced biases, PCR products were purified with Agencourt AMPure XP SPRI magnetic beads (Beckman Coulter, Tokyo, Japan). The DNA quantity was measured with the NanoDrop 2000 spectrophotometer (Thermo Scientific, Waltham, MA, United States).

Amplicon pools for all samples were paired-end sequenced (2 × 250) on an Illumina MiSeq platform at Magigen Biotechnology (Guangzhou, China). The raw data for each pool of samples were separately trimmed and *de novo* assembled in a unique file using CLC Genomics Workbench (Version 6.0.2, CLC Bio, Denmark) alignment and annotation tools. We removed sequences if they (1) contained more than one ambiguous nucleotide; (2) lacked a complete barcode and primer at one end; or (3) were shorter than 200 bp after removal of the barcode and primer. The overlap settings for this assembly were a mismatch cost of 2, an insert cost of 3, a minimum contig length of 200 bp, a similarity of 0.8, and a trimming quality score of 0.05. The sequences were clustered at 97% similarity cutoff into operational taxonomic units (OTUs). The representative sequence of each phylotype was aligned against the SILVA database (release 132) with a confidence threshold of 80%.

### Inference of Interspecific Interactions

We constructed the interspecific interactions of bacterial OTUs by the CoNet (v.1.1.1.beta) plugin in Cytoscape software (v3.5.1), as previously described (Faust and Raes, 2016). To reduce noise and false-positive predictions, network inclusion was restricted to OTUs that appeared in at least 80% of samples. In each network, pairwise associations between OTUs were simultaneously identified by an ensemble of correlations (Pearson and Spearman coefficients) and distance metrics (Kendall distance, Bray-Curtis distance, and Kullback-Leibler dissimilarity measures). The initial 500 top- and bottom-ranking edges were kept in the network. A total of 1,000 permutation scores and 1,000 bootstrap scores were calculated for each edge and each measure of association. The measure-specific *p*-values from multiple interaction metrics were merged using the Simes method (Sarkar and Chang, 1997), and false discovery rate correlation was performed using the Benjamini-Hochberg multiple procedure (Benjamini and Hochberg, 1995). A set of properties was calculated by the “igraph” package, including the number of nodes and edges, average degree (avgK), density, average path distance (APD), and modularity (Zhou et al., 2012). To test the significance of networks, 1000 Erdös–Rényi random networks were obtained with the “igraph” R package, which had the same numbers of nodes and edges as the empirical networks.

### Statistical Analysis

All analyses and visualizations were mainly performed by ‘‘picante,’’ ‘‘cowplot,’’ ‘‘dplyr,’’ ‘‘factoextra,’’ ‘‘ggplot2,’’ ‘‘HiveR,’’ ‘‘igraph,’’ ‘‘mvpart,’’ ‘‘reshape2,’’ and ‘‘vegan’’ in the R environment (version 3.2.2^1^) and the RStudio interface (version 1.1.463). To explore the major characteristics of aquatic physico-chemical parameters, principal component analysis (PCA) and hierarchical clustering analysis were utilized. A Kruskal-Wallis test was used to identify differences in individual parameters among distinct lakes. Data sets for multivariate statistical analyses, diversity estimates and interspecific interaction construction were rarefied according to the lowest numbers of reads among all samples (59,188 reads). The richness, Shannon, and Simpson indices were representative of the alpha-diversity of bacterial communities. OTUs were binned into the “core community” if they were present in 100% (referred to as the Core100) and 80% (Core80) of all samples. To statistically test the difference of bacterial communities among distinct lakes, non-metric multidimensional scaling (NMDS) and PerMANOVA were applied based on Bray-Curtis dissimilarity with 999 permutations. To compare the significant differential abundance of bacterial communities between pairwise lakes, Statistical analysis of metagenomic profiles (STAMP) was applied at the phylum, class, order, family and genus levels (Parks and Beiko, 2010), and differential expression analysis (Deseq2) was applied at the OTU level (Love et al., 2014). To reveal the importance of physicochemical parameters in predicting the bacterial assemblages, multiple regression tree (MRT) analysis which characterizes non-linear relationship was used. Further, to quantify the balance of stochastic and deterministic processes in governing the community turnover, we used a null model approach (999 randomizations) described before (Stegen et al., 2013; Dini-Andreote et al., 2015). Phylogenetic signal was primarily tested to determine whether the phylogenetic turnover can be used to infer the ecological assembly processes. As previously suggested (Dini-Andreote et al., 2015), a βNTI > 2 indicates that variable selection is the dominant assembly process governing the bacterial communities, while a βNTI < −2 indicates that homogeneous selection plays a leading role. |βNTI| < 2 indicates the absence of selection, and a greater influence of stochastic processes, such as dispersal and/or drift. Niche breadth was assessed as the average niche breadth of all members appearing in the community (Jiao et al., 2020).

### Data Deposition

The data presented in the study are deposited in the Genome Sequence Archive in BIG Data Center, Beijing Institute of Genomics, Chinese Academy of Sciences, accession number CRA004510.

## Results

### Aquatic Environmental Characteristics

**FIGURE 2:**
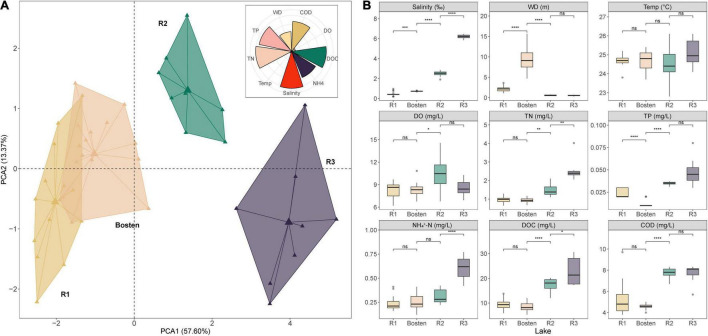
The main environmental characteristics among the four lakes based on salinization and nutrient enrichment gradients. **(A)** PCA plot of the sampling sites for all lakes. The rose diagram shows the contributions of individual parameters to the total environmental variation. **(B)** Boxplot comparison of the aquatic environments among the four lakes based on the simulated salinization and nutrient enrichment gradients, using the Kruskal-Wallis test to examine the significance levels of the differences. Significance levels: ns: *p*> 0.05; *p < 0.05; **p < 0.01; ***p < 0.001. WD, water depth; Temp, temperature; DO, dissolved oxygen; TN, total nitrogen; TP, total phosphorus; NH_4_^+^-N, ammonia; DOC, dissolved organic carbon; COD, chemical oxygen demand.

According to the PCA, 89.88% of the variation in the aquatic environment was explained by the first four principal components, among which the first two accounted for 57.60 and 13.37%, respectively. Specifically, salinity (16.44%) was the parameter that contributed the most, followed by TN (16.06%), DOC (15.45%), and TP (14.80%). The PCA results also indicated that these four lakes were significantly heterogeneous, although there was overlap between Lake R1 and Lake Bosten (Figure 2A). This indication was further supported by a permutational multivariate analysis of variance test, which revealed that the aquatic environment was significantly different between each pair of lakes (Supplementary Table 1).

Specifically, Lake Bosten was significantly deeper than Lake Acacia (9.58 m vs. 1.35 m, *p* < 0.001). Temperature and DO were comparable in all lakes oscillating around 24.70°C and 8.73 mg/L, respectively. Salinity differed significantly between all pairs of lakes (Kruskal-Wallis test, *p* < 0.01), with Lake R1 having a salinity of 0.47‰ (freshwater), Lake Bosten having a salinity of 0.74‰ (low-brackish water), Lake R2 having a salinity of 2.48‰ (high-brackish water), and Lake R3 having a salinity of 6.22‰ (brine water). Except for TP, the concentrations of nutrients (represented by TN, NH_4_^+–^N, DOC, and COD) were similar between Lake R1 and Lake Bosten. Particularly, these nutrients were significantly higher in Lake R2 and Lake R3 (*p* < 0.05). Furthermore, nutrients except for TP and COD were much higher in Lake R3 than in Lake R2 (*p* < 0.05). Collectively, there was an increasing trend of salinity and nutrient concentration from Lake R1 to Lake Bosten, R2, and R3, suggesting simultaneous salinization and nutrient enrichment (Figure 2B).

### The Bacterial Communities and Their Underlying Ecological Processes

**FIGURE 3:**
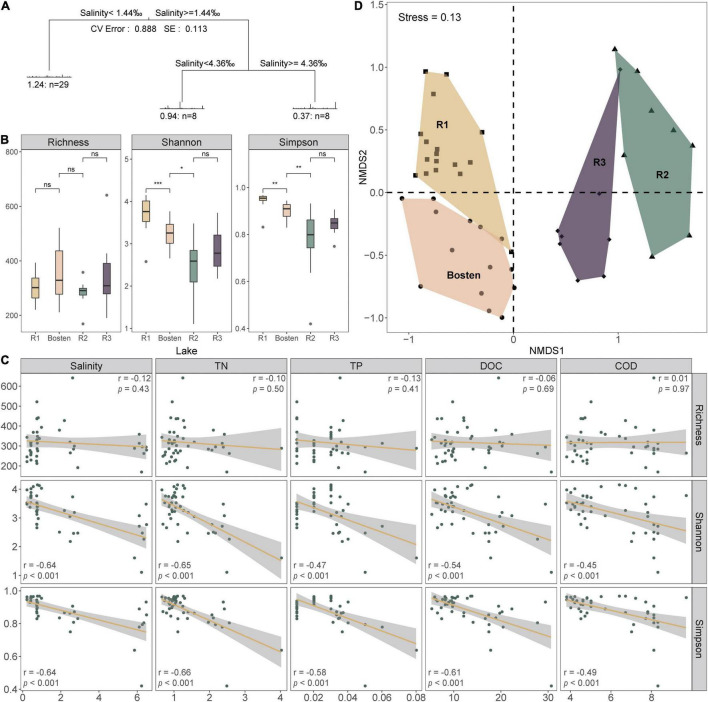
The diversity of the bacterial communities and their driving forces. **(A)** Multivariate regression tree of bacterial community classification. Each leaf in the tree shows the number of bacterial communities, and drivers of their structures are specified along the branches of the tree. CV Error was calculated as the percentage of variance explained by the tree, and SE presents the standard error from 10-fold cross validation. **(B)** The alpha-diversity of the bacterial communities for the four lakes based on the simulated salinization and nutrient enrichment gradients. Significance levels: ns: *p*-value > 0.05; *p-value < 0.05; **p-value < 0.01; ***p-value < 0.001. **(C)** The relationships between the alpha-diversity and main environmental variables. **(D)** Non-metric multidimensional scaling (NMDS) ordination of bacterial communities based on Bray-Curtis dissimilarity.

In average, the sequencing has produced 77,823 high-quality reads per site. In total, 2,645 OTUs were obtained among which 1,107, 1,382, 831, and 1,172 OTUs appeared in Lakes R1, Bosten, R2, and R3, respectively. Rarefaction analysis suggested that the observed OTUs approached an asymptote in each lake (Supplementary Figure 1). The core community was clearly higher in Lake R2. For instance, the Core100 community gradually increased from 3.43 to 5.07% to 8.30% from Lake R1 to Bosten to Lake R2 and then decreased back to 3.75% in Lake R3. Similarly, the Core80 community also increased from 9.62 to 11.56% to 16.00%, and then decreased to 11.77%. Measures of within-sample diversity exhibited a decreasing trend from Lake R1 to Lake Bosten, R2, and R3. Although richness was comparable in the four lakes (approximately 320 OTUs per lake) (Figure 3B), the Shannon and Simpson indices decreased significantly from Lake R1 to Lake Bosten and to R2 (Kruskal-Wallis test, *p* < 0.05), and then increased slightly in Lake R3 with non-significant differences (*p* > 0.05) (Figure 2B). Furthermore, both Shannon and Simpson indices were negatively associated with salinity, TN, TP, DOC, and COD (all *p* < 0.001, Figure 3C).

**FIGURE 4:**
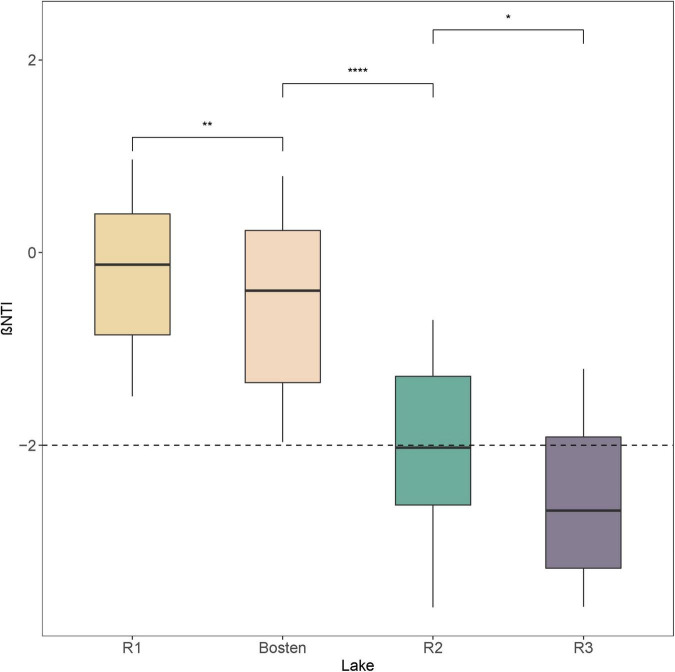
The beta nearest taxon index (βNTI) of bacterial communities among the four lakes. Significance levels: *p-value < 0.05; **p-value < 0.01; ***p-value < 0.001.

The MRT model was applied to explore the relationships between bacterial communities and environmental parameters, which explained 88.8% of the total variation for the bacterial communities (Figure 3A). This model revealed that salinity was the primary predictor of bacterial community composition. Specifically, the bacterial communities within the four lakes were split into two clusters by a salinity of 1.44‰: Cluster 1 (Lakes R1 and Bosten) and Cluster 2 (Lakes R2 and R3). The bacterial communities of Lake R2 and Lake R3 within Cluster 2 were further split by a salinity of 4.36‰. NMDS was also performed to investigate the separation pattern of the bacterial communities. The sampling sites were separated across from left to right (Figure 3D), suggesting that the largest source of community variation was salinity. Consistently, PerMANOVA also indicated that the bacterial communities were significantly different among distinct salinity levels (Supplementary Table 2, all *p* < 0.001). Furthermore, we examined the relative contribution of deterministic and stochastic processes to the bacterial communities within individual lakes (Figure 4). The βNTI was comparable between Lakes R1 and Bosten, ranging from 0 to −2, which suggested that the bacterial communities in Lakes R1 and Bosten were shaped by stochastic processes. In contrast, the βNTI significantly decreased to less than −2 in Lakes R2 and R3, highlighting deterministic processes were responsible for the bacterial communities.

**FIGURE 5:**
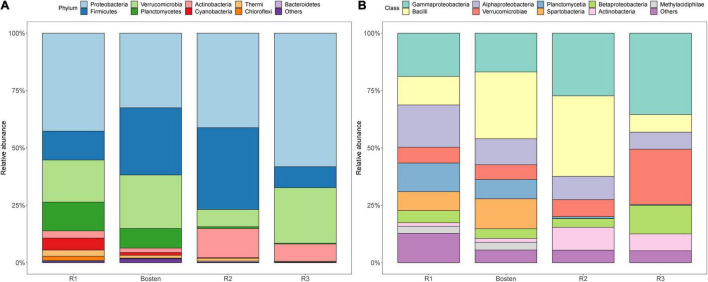
The bacterial community compositions of four lakes at the phylum **(A)** and class levels **(B)**.

**FIGURE 6:**
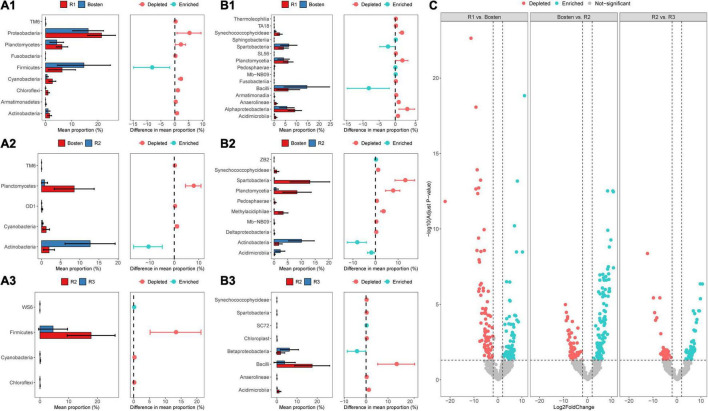
The significantly changed bacterial groups from Lake R1 to Lake Bosten, R2, and R3 (*P* < 0.05). **(A1–A3)** For the phylum level, **(B1–B3)** for the class level, **(C)** for the OTU level.

**FIGURE 7:**
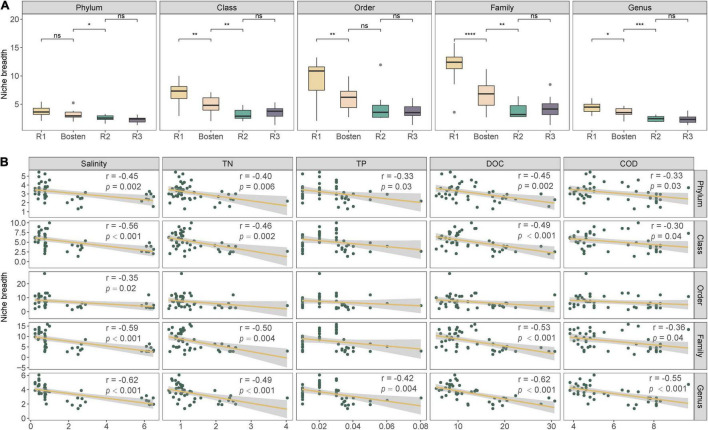
The niche breadth of bacterial communities among the four lakes **(A)** and their relationships with environmental parameters **(B)**. Significance levels: ns: *p*-value > 0.05; *p-value < 0.05; **p-value < 0.01; ***p-value < 0.001.

At the phylum level (Figure 5), the bacterial communities of the four lakes were dominated by Proteobacteria (43.65%), followed by Firmicutes (21.66%) and Verrucomicrobia (18.34%). Planctomycetes prevailed in both Lakes R1 and Bosten but was rare in Lakes R2 and R3 (10.53 vs. 0.57%); in contrast, Actinobacteria was less dominant in Lakes R1 and Bosten but significantly increased in Lakes R2 and R3 (2.55 vs. 10.10%). At the finer class level (Figure 4), Gammaproteobacteria (24.61%) followed by Bacilli (21.09%), Alphaproteobacteria (11.83%), and Verrucomicrobiae (11.23%), was dominant in the four lakes. In particular, Gammaproteobacteria and Verrucomicrobiae gradually increased from Lake R1 to Lakes Bosten, R2, and R3, while Alphaproteobacteria and Bacillia gradually decreased, showing a hump-shaped trend that peaked in Lake R2. At the phylum and class levels, the STAMP analysis revealed that the significantly changed members declined from Lake R1 to Lakes Bosten, R2, and R3 (Figures 6A,B). At the OTU level, the DESeq2 differential abundance analysis also indicated that the number of significantly changed OTUs was comparable between the comparisons of Lakes R1 and Bosten and Lakes Bosten and R2, but clearly declined from Lake R2 to Lake R3 (Figure 6C). Moreover, the niche breadth of bacterial communities similarly showed a decreasing trend from Lake R1 to Lakes Bosten, R2, and R3 (Figure 7A), which was negatively associated with salinity and nutrient species (all *p* < 0.05, Figure 7B). Together, these observations highlighted decrease in community variability along the salinization and nutrient enrichment gradients.

### The Interspecific Interactions of the Bacterial Communities

**FIGURE 8:**
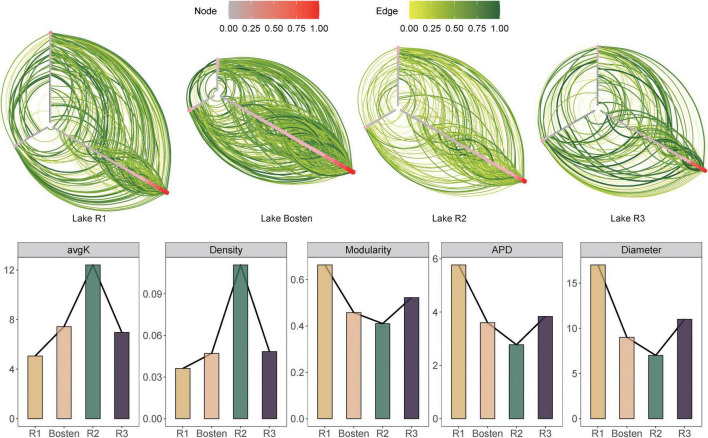
Hive plot of interspecific interactions for the bacterial communities of the four lakes. To visually compare the individual networks, nodes (in red) and weight of edges (in green) were scaled to 1.

The OTU-specific networks exhibited unique interspecific interactions of bacterial communities within the four lakes (Figure 8). The reliability and non-randomness of the four empirical networks were verified by comparison with a random network (Supplementary Table 3). These empirical networks had an average node number of 140 and edge number of 539, with the network of Lake R2 containing the fewest (64) nodes but the most (701) edges. Within the four networks, the proportion of positive interactions far exceeded that of negative interactions (87.36 vs. 12.64%). Notably, the proportion of positive interactions gradually increased from 73.08 to 93.58% from freshwater Lake R1 to brine Lake R3. In terms of network topologies, the average degree and density showed a hump-shaped curve along gradients of salinization and nutrient enrichment, ranging from 6.25 to 12.41 to 6.97 and from 0.05 to 0.11 to 0.05, respectively. In contrast, modularity, density, and average path distance showed a sunken curve, ranging from 0.54 to 0.46 to 0.66, from 0.04 to 0.11 to 0.04, and from 5.77 to 2.78 to 3.84, respectively.

## Discussion

In the twenty-first century, the ongoing climate change has increasingly impacted lake ecosystems in arid and semiarid areas. To build an understanding of the adaptive strategy of bacterial communities in lake ecosystems, it is essential to recognize the responses of bacterial communities to two climate-related disturbances: salinization and nutrient-enrichment. By a space-for-time substitution approach, our hypothesis was confirmed that climate-related disturbances induced the profound turnover of bacterial communities, with salinization exerting more effects than nutrient-enrichment. Evidently, salinization not only determines the diversity and composition of bacterial communities, but also alters their interspecific interactions. Our work provides new insights into the implications of future climate change for lake ecosystems.

### Salinization Induces Convergent Response of Bacterial Communities

The current results showed that salinity was the primarily variable affecting the aquatic bacterial communities, which was supported by significant differences in bacterial communities between habitats with different salinity levels, as well as the prediction of the MRT model (Figure 2). Consistently, this implication is also in line with the findings of previous studies in arid and semiarid areas (Bernhard et al., 2005; Tandon et al., 2020; Tang et al., 2021). Regarding bacterial community composition, Gammaproteobacteria, known to prefer saline environments, gradually became the dominant group in brine Lake R3, whereas Alphaproteobacteria and Bacillia, which are competitive in freshwater, significantly decreased from freshwater Lake R1 to brine Lake R3 (Figure 5). The reason is that salinization enhances osmotic pressure which decreases the survival cost of halophilic bacteria to acquire nutrients while comparatively increases that cost of halophobic bacteria (Newton et al., 2011). Additionally, salinization leads to a significant loss in diversity (Figure 2), suggesting an adverse effect on bacterial communities (Liu et al., 2013; Tang et al., 2021). This effect could also be reflected by the significant negative association between salinization and niche breadth (Figure 7). It is noteworthy that nutrients seem to alleviate the adverse effect of salinization. For instance, a nutrient-rich environment promotes the ability of bacteria to build proteins to cope with high salinity (Yue et al., 2019). Thus, nutrients are expected to promote the niche expansion of bacterial communities, as well as species diversification (Joy, 2013; Kiersztyn et al., 2019). However, this expectation contradicts our result that community diversity and niche breadth were negatively associated with the enrichment of nitrogen, phosphorus, and carbon (Figure 6). Therefore, we speculate that the adverse effect of salinization may overwhelm the beneficial effect of nutrient enrichment on bacterial communities in arid and semiarid areas. In other words, salinization decreases the nutrient acquisition ability of bacterial communities, even if there are ample available nutrients in lake ecosystems.

Our study further revealed convergent response of bacterial communities along the salinization gradient, which was inferred from less variable communities from freshwater Lake R1 to brine Lake R3 (Figure 2). Here, we ascribed this convergent response to the great influence of homogeneous selection via niche overlap induced by salinization. According to the decreasing βNTI (Figure 3), the bacterial communities were expected to be increasingly controlled by deterministic processes, which commonly introduce two niche-based types of selections: homogeneous selection and variable selection (Dini-Andreote et al., 2015; Gu et al., 2021). Generally, niche overlap triggers homogenous selection, leading to a low level of community variation, whereas niche expansion triggers variable selection, leading to a high level of variation (Nunoura et al., 2016; Cabrera-Mora et al., 2019; Morganti et al., 2020). Nutrient enrichment affords opportunities for the niche expansion of bacterial communities (Cui et al., 2020); however, the adverse effect of salinization actually decreases their realized niche, as our results showed gradually decreasing niche breadth from freshwater Lake R1 to brine Lake R3 (Figure 6). Consequently, niche overlap leads to convergent response of bacterial communities.

### Moderately High-Brackish Salinity Destabilizes Bacterial Communities

It has long been acknowledged that bacteria do not live in isolation but form an ecological interaction network, which consist of mutualistic and antagonistic interactions (Faust and Raes, 2012). Generally, bacterial interactions are highly asymmetric. Due to the roles of supplementing others’ growth at the species level and facilitating robust coexistence at the community level, mutualistic interactions commonly dominate antagonistic interactions in bacterial communities in various habitats (Magnusdottir et al., 2017; Stone, 2020; Sun et al., 2020). This is in agreement with the results of our study, in which positive interactions accounted for approximately 87.36% of the interactions within the four networks. Crucially, however, the balance of mutualistic and antagonistic interactions in bacterial communities is likely environment-dependent (Yu et al., 2020). The previous stress gradient hypothesis (SGH) predicts that a harsh environment intensifies antagonistic interactions, while a benign environment favors mutualistic interactions (Bertness and Callaway, 1994). However, the current network showed that positive interaction clearly increased (from 73.33 to 92.39%) with salinization. Our observation is not an exceptional case: for instance, positive interactions increased from 51.1% in non-saline soil network to 98.1% in a saline soil network (Ma et al., 2020). Thus, these results are not in accordance with the prediction of the SGH. As we discussed above, salinization leads to overlapping niches by decreasing the nutrient availability for bacterial communities; however, bacteria do not simply passively inhabit harsh environments but actively interact with each other to promote their fitness. On the one hand, they often attempt to outcompete other bacteria to reduce competition in terms of limited environmental resources (Baishya and Wakeman, 2019). For instance, to cope with the limitation of essential nutrients, *Pseudomonas aeruginosa* can secrete reactive oxygen species (ROS) to eliminate competitive microorganisms such as *Burkholderia cepacia* (Tomlin et al., 2001). On the other hand, they can also take advantage of others’ waste products via metabolite exchange such as cross-feeding, co-colonization, and co-aggregation (Faust and Raes, 2012). For instance, yeast colonies prefer the exchange of extracellular metabolites (histidine, leucine, uracil, and methionine) to compensate for the progressive loss of prototrophy (Campbell et al., 2015). Thus, such metabolic processes are most likely responsible for the active transition from decreasing antagonistic interactions to increasing mutualistic interactions as environments become more saline.

Furthermore, our study showed that salinization destabilized the bacterial communities, which was underpinned by two lines of evidence. First, salinity significantly decreased the diversity of the bacterial community from freshwater to brine lake (Figure 2B). Although recent studies have argued that functional diversity is more directly related to community stability than pure species diversity, the diversity-stability relationship theoretically posits that community diversity also gives rise to ecological stability (Coyte et al., 2015). On the one hand, higher diversity increases the odds that at least some species will respond differentially to and cope with variable perturbations. On the other hand, greater diversity also increases the odds that the bacterial community is functionally redundant by harboring species that are capable of replacing other species (McCann, 2015). Second, decreasing antagonistic interactions may exert a destabilizing effect on complex bacterial communities. In woven coupling communities, even though facilitative mechanisms promote cooperation in which one species benefits from another’s existence and replication, they do not equate to stability. A possible reason is that mutualism leads to unbounded positive feedbacks, which create instability (Coyte et al., 2015). To dampen these unbounded positive feedbacks, antagonistic interactions limit the benefits that a focal species receives from its mutualist partners (Qian and Akcay, 2020) and thus increase community stability (Yu et al., 2020). Induced by salinization, decreasing biodiversity and increasing mutualistic interactions collectively destabilize bacterial communities.

Although increasing salinity destabilized the bacterial communities, further topological properties indicated that the bacterial communities were more destabilized in high-brackish Lake R2 than in brine Lake R3. The first source of support is that the average degree and density of community networks were more than twice as high in Lake R2 compared with Lake R3 (12.41 vs. 6.96 and 0.11 vs. 0.05, respectively), suggesting that the bacterial assemblage was more tightly connected in the former lake. Simultaneously, the average path distance, which is biologically regarded as the length of the food chain flow of nutrients and energy, was shorter in Lake R2 than in Lake R3 (2.77 vs. 3.84). Together, these topologies demonstrated the most interactive communities in the high-brackish environment. Intuitively, close interactions lead to stable and robust communities; however, recent theories have challenged this intuition (Neutel et al., 2002; de Vries et al., 2018). Recent studies suggested that once a given species is stimulated in a tightly interactive community, closely dependent species would respond in sensitive rise or downfall, such that the bacterial community would be vulnerable to disturbance (Coyte et al., 2015). Another source of support is the lower modularity of the bacterial communities in Lake R2 than Lake R3 (0.46 vs. 0.52), which measures the extent to which a network is separated into modules. Within the highly separated bacterial communities, the effect of environmental disturbance could be strained in a single cohesive module without transferring to neighboring ones. Therefore, less modularity implies less resistance of bacterial communities.

## Conclusion

First, this study reveals that salinity rather than nutrient is the most variable in arid and semiarid areas in Xinjiang. Second, there are less diverse and less variable communities from freshwater to brine lakes, highlighting that salinization induces convergent response of bacterial communities. Third, the topologies of interspecific interactions along the salinity implied that salinization destabilized the bacterial communities. On particular, the bacterial communities were more unstable in the high-brackish lake than in the brine lake. Our findings have important implications for understanding how complex aquatic microbial communities respond to climate-related disturbances in arid and semi-arid area, which is pivotal for advancing our understanding of the evolutionary strategies of lake ecosystems.

## Data Availability Statement

The datasets presented in this study can be found in online repositories. The names of the repository/repositories and accession number(s) can be found below: https://bigd.big.ac.cn/gsa, CRA004510.

## Author Contributions

YH, GG, and BQ conceived and designed the experiments. XJ and KS performed sample collection. YH and XT analyzed the data. YH wrote the manuscript. All authors contributed to the article and approved the submitted version.

## Conflict of Interest

The authors declare that the research was conducted in the absence of any commercial or financial relationships that could be construed as a potential conflict of interest.

## Publisher’s Note

All claims expressed in this article are solely those of the authors and do not necessarily represent those of their affiliated organizations, or those of the publisher, the editors and the reviewers. Any product that may be evaluated in this article, or claim that may be made by its manufacturer, is not guaranteed or endorsed by the publisher.

## Footnotes

1
http://www.r-project.org
