# Supplementary figures and images for: Convergency and Stability Responses of Bacterial Communities to Salinization in Arid and Semiarid Areas: Implications for Global Climate Change in Lake Ecosystems
Source: Front Microbiol. 2022 Jan 4;12:741645. doi: 10.3389/fmicb.2021.741645 (PMC8764409; doi:10.3389/fmicb.2021.741645)

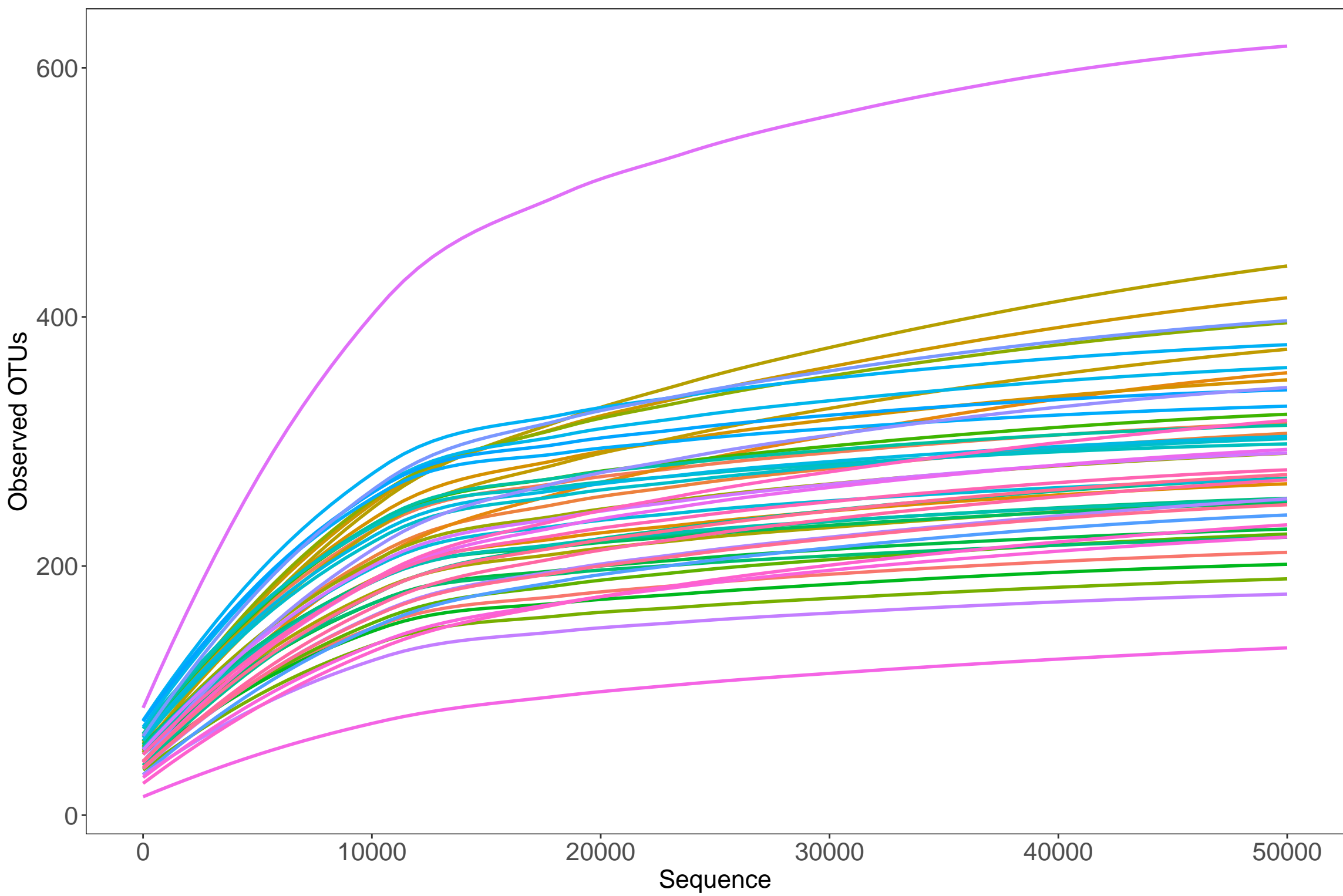

Supplement: Supplementary file 1 [file Data_Sheet_1.PDF]

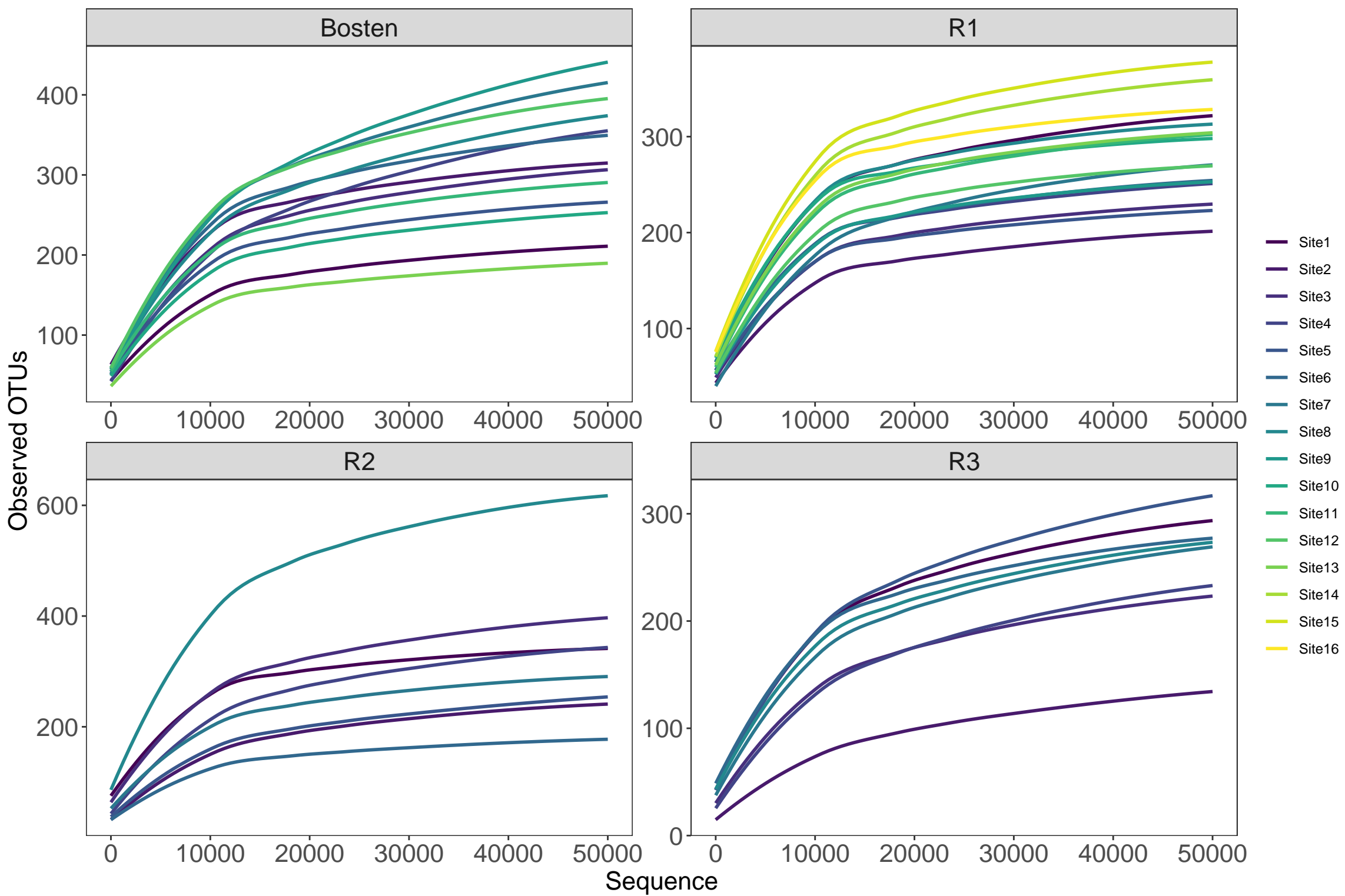

Supplement: Supplementary file 2 [file Image_1.pdf]
