# Supplementary material for: Convergency and Stability Responses of Bacterial Communities to Salinization in Arid and Semiarid Areas: Implications for Global Climate Change in Lake Ecosystems
Source: Front Microbiol. 2022 Jan 4;12:741645. doi: 10.3389/fmicb.2021.741645 (PMC8764409; doi:10.3389/fmicb.2021.741645)
Supplement: Supplementary file 3 [file Table_1.DOCX]

**Convergency and stability responses of bacterial communities to salinization in arid and semiarid areas:** **Implications for global climate change in lake ecosystems**

Yang Hu^1^, Xingyu Jiang^1^, Keqiang Shao^1^, Xiangming Tang^1^, Boqiang, Qin^1^, and Guang Gao^1,^^[[1]](#footnote-1)^*

^1^Taihu Laboratory for Lake Ecosystem Research, State Key Laboratory of Lake Science and Environment, Nanjing Institute of Geography and Limnology, Chinese Academy of Sciences, Nanjing 210008, China;

Table S1 Results of PERMANOVAs based on Euclidean distance matrices derived from log-transformed environmental data

| group | MS | F.Model | R2 | *P* |
| --- | --- | --- | --- | --- |
| Acacia_R1 vs Acacia_R2 | 18.88 | 46.42 | 0.69 | < 0.001*** |
| Acacia_R1 vs Acacia_R3 | 47.14 | 124.94 | 0.86 | < 0.001*** |
| Acacia_R1 vs Bosten | 7.11 | 23.55 | 0.76 | 0.016* |
| Acacia_R2 vs Acacia_R3 | 5.07 | 17.46 | 0.59 | 0.002** |
| Acacia_R2 vs Bosten | 17.11 | 80.99 | 0.81 | < 0.001*** |
| Acacia_R3 vs Bosten | 42.16 | 236.05 | 0.93 | < 0.001*** |

Table S2 Results of PERMANOVAs based on Bray-Curtis distance matrices

| group | MS | F.Model | R2 | *P* |
| --- | --- | --- | --- | --- |
| Acacia_R1 vs Acacia_R2 | 2.78 | 19.091 | 0.78 | 0.001*** |
| Acacia_R1 vs Acacia_R3 | 2.23 | 16.67 | 0.44 | 0.001*** |
| Acacia_R1 vs Bosten | 1.48 | 9.65 | 0.26 | 0.001*** |
| Acacia_R2 vs Acacia_R3 | 1.07 | 6.95 | 0.37 | 0.002** |
| Acacia_R2 vs Bosten | 2.32 | 13.32 | 0.41 | 0.001*** |
| Acacia_R3 vs Bosten | 1.30 | 8.06 | 0.30 | 0.001*** |

Table S3 The significant test between empirical networks and random networks

| Network topologies | Lake | | | |
| --- | --- | --- | --- | --- |
| *Empirical Networks* | R1 | Bosten | R2 | R3 |
| Total nodes | 159 | 141 | 113 | 145 |
| Total edges | 591 | 357 | 701 | 505 |
| Average degree (avgK) | 7.43 | 5.06 | 12.41 | 6.97 |
| Average path distance (APD) | 3.60 | 5.77 | 2.77 | 3.84 |
| Modularity | 0.66 | 0.46 | 0.42 | 0.52 |
| Graph density | 0.05 | 0.04 | 0.11 | 0.05 |
| Graph diameter | 17 | 9 | 7 | 11 |
| *Random Networks* |  |  |  |  |
| Average path distance (APD) | 3.126±0.014 | 3.624±0.010 | 2.301±0.019 | 3.248±0.024 |
| Modularity (M) | 0.241±0.001 | 0.308±0.006 | 0.232±0.004 | 0.308±0.003 |

1. *Corresponding author: Guang Gao, Nanjing Institute of Geography and Limnology, Chinese Academy of Sciences, 73 East Beijing Road, Nanjing 210008, China

   E-mail: guanggao@niglas.ac.cn

   Tel: (+86) 25 86882187 [↑](#footnote-ref-1)
